# Picobirnaviruses in the Human Respiratory Tract

**DOI:** 10.3201/eid1809.120507

**Published:** 2012-09

**Authors:** Saskia L. Smits, Marije van Leeuwen, Claudia M.E. Schapendonk, Anita C. Schürch, Rogier Bodewes, Bart L. Haagmans, Albert D.M.E. Osterhaus

**Affiliations:** Erasmus Medical Center, Rotterdam, the Netherlands (S. Smits, C.M.E. Schapendonk, A.C. Schürch, R. Bodewes, B.L. Haagmans, A.D.M.E. Osterhaus);; and Viroclinics Biosciences BV, Rotterdam (S.L. Smits, M. van Leeuwen, A.D.M.E. Osterhaus)

**Keywords:** human, picobirnavirus, respiratory tract, PCR, viruses

**To the Editor:** Picobirnaviruses (family *Picobirnaviridae*) are nonenveloped, double-stranded RNA viruses of vertebrates with a bisegmented genome. Segment 1 (2.2–2.7 kb) encodes the capsid protein, and segment 2 (1.2–1.9 kb) encodes the RNA-dependent RNA polymerase. On the basis of sequence diversity in segment 2, picobirnaviruses are classified into 2 genogroups (*1*–*4*). Picobirnaviruses have been detected in fecal samples from humans with and without gastroenteritis; in patients co-infected with known enteric pathogens, including rotaviruses, caliciviruses, and astroviruses (*1*,*4*); and in a wide range of animals, such as pigs, calves, dogs, monkeys, and snakes. The pathogenicity of picobirnaviruses largely remains to be determined, but studies in immunocompromised persons suggest that picobirnaviruses may be opportunistic enteric pathogens (*5*,*6*).

Recently, we identified picobirnaviruses in the respiratory tract of pigs in Asia, and this identification expanded the knowledge on the tropism and host range of picobirnaviruses (*7*). No respiratory or other clinical signs were observed in these pigs at the time of sampling, making it unclear whether picobirnaviruses are indeed respiratory pathogens (*7*). To determine whether picobirnaviruses could also be present in the human respiratory tract, we performed a diagnostic genogroup I picobirnavirus PCR, with degenerated primers, that targeted the RNA-dependent RNA polymerase coding region (*1*,*4*,*8*) on 309 bronchoalveolar lavage specimens collected from 309 patients with respiratory disease of unknown origin in the Netherlands during 2003–2006. (All study procedures were performed in compliance with relevant laws and institutional guidelines and in accordance with the Declaration of Helsinki.)

Samples from 3 patients were confirmed by sequencing to be positive for genogroup I picobirnaviruses. To determine genetic relationships between human genogroup I picobirnaviruses from the respiratory tract and genogroup I picobirnaviruses detected in wastewater and in human and porcine fecal samples, we constructed a phylogenetic tree on the basis of a ≈165-nt fragment of the RNA-dependent RNA polymerase gene as described (*8*) (Figure). Before tree construction, 75 groups were created from the ≈300 available picobirnavirus sequences by using FastGroup II (*10*). Because the average pair-wise Jukes-Cantor distance was 0.46, a neighbor-joining tree was created by using the Jukes-Cantor model, with a bootstrap replication of 1,000 (Figure). One of the 3 genogroup I picobirnavirus sequences found in this study, PBVI/Homo sapiens/VS2000057/2003, showed <95% sequence identity with previously described picobirnavirus sequences and is shown as a separate branch in the phylogenetic tree. The genogroup I picobirnavirus nucleotide sequences from the respiratory tracts of persons in the Netherlands showed 58% to 97% similarity with each other. They belonged to different phylogenetic clades and did not group with other picobirnaviruses according to year of isolation or host species.

**Figure:**
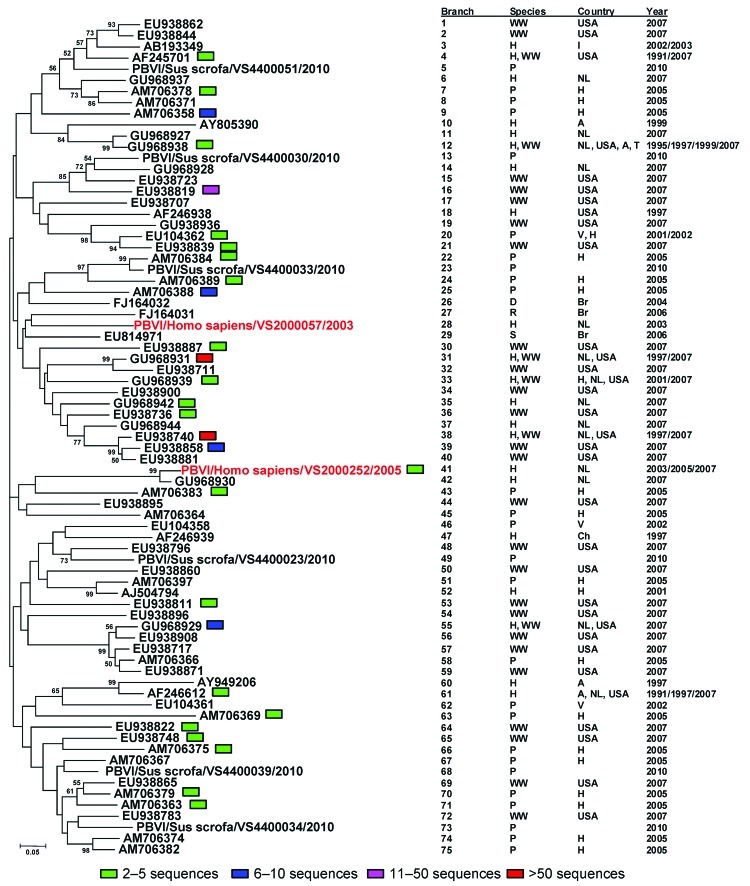
Neighbor-joining (Jukes-Cantor model) phylogenetic tree of an ≈165-bp fragment of the genogroup I picobirnavirus RNA-dependent RNA polymerase gene from known human, porcine, and wastewater genogroup I picobirnaviruses and newly characterized genogroup I picobirnaviruses (sequences are available on request) from the human respiratory tract. Each branch represents a sequence or group of sequences (95% identical with gaps) indicated by the presence of a colored block. Every branch corresponds to the adjacent table, which explains the source, year of isolation, and location of the virus variants. Reference sequences from previously published studies are identified by their GenBank accession numbers. The designations for picobirnaviruses from various species are designated as follows: P, porcine; WW, wastewater; D, dog; S, snake; R, rat; H, human; The different countries of isolation are designated as follows: A, Argentina; Br, Brazil; Ch, China; H, Hungary; I, India; NL, the Netherlands; USA, United States of America; T, Thailand; V, Venezuela. The sequences from the newly identified human respiratory picobirnaviruses in this study comply with recent nomenclature proposals (*9*) and are indicated in red.

In conclusion, the identification of new picobirnaviruses in respiratory tract samples from pigs (*7*) prompted us to look for the presence of picobirnaviruses in the respiratory tracts of humans. Genogroup I picobirnaviruses could be identified in some of the bronchoalveolar lavage specimens obtained from patients with unexplained respiratory disease in the Netherlands. This observation expands our knowledge of picobirnaviruses in humans and provides a clear example of how epidemiologic baseline information on virus host range and tropism in animals may provide indications for the presence of similar viruses in the same organ system of humans. To clarify the epidemiology and pathogenicity of picobirnaviruses in humans, additional surveillance should be carried out in persons with and without respiratory and enteric disease.
